# 4030 Development of a Falls-Prevention Self-Management Plan for Community Dwelling Older Adults

**DOI:** 10.1017/cts.2020.381

**Published:** 2020-07-29

**Authors:** Jennifer L. Vincenzo, Leanne L. Lefler, Susan Kane Patton, Jeanne Wei

**Affiliations:** 1 University of Arkansas Translational Research Institute; 2 University of Arkansas; 3 University of Arkansas for Medical Sciences

## Abstract

OBJECTIVES/GOALS: 1. To determine older adults’ opinions on content that is valuable for inclusion in a falls-prevention self-management plan. 2. To determine older adults’ recommendations of mode(s) to promote adherence to falls prevention recommendations. METHODS/STUDY POPULATION: On-on-one semi structured interviews with older adults are ongoing to determine their opinions on content for inclusion in a falls-prevention self-management plan and recommendations for mode of delivery. As in our prior investigations, we used the theoretical constructs of the health belief model to develop our questions. Interviews will be recorded and transcribed. Data will be entered into MAXQDA12 and coded. Concurrent data collection and analysis will continue until theoretical saturation of themes are achieved. Through this iterative process, we will identify content and mode of delivery for a falls- prevention self-management plan for implementation with older adults. RESULTS/ANTICIPATED RESULTS: We anticipate we will have conducted enough interviews to achieve data saturation by February, 2020. We expect the results of this qualitative investigation to guide the development of a falls-prevention self-management plan that includes targeted implementation and adherence strategies deemed acceptable and feasible for use among older adults following community-based falls-risk screenings. DISCUSSION/SIGNIFICANCE OF IMPACT: Falls are the leading cause of morbidity and mortality among older adults. Adherence to falls prevention among older adults is poor, even among those that voluntarily seek out recommendations. The results of this study will assist with development and pilot testing of a falls-prevention self-management plan to assist older adults to adhere to recommendations.

